# Personal

**Published:** 1892-04

**Authors:** 


### Personal.

---

DR. E. E. MONTGOMERY, having resigned the obstetrical chair in the Medico-Chirurgical College of Philadelphia, will hereafter devote himself entirely to teaching in the gynecological department of that college.

---

DR. JOSEPH PRICE, of Philadelphia, has been for a few years past the physician in charge of the Preston Retreat, which is one of the largest maternities in this country. During his service, now something like four years, there have been 1,050 confinements in that institution, including one Porro-Cesarian section, without a death. This is one of the most satisfactory records that has been made in any maternity, and speaks volumes for the modern methods of hygiene, sanitation, and obstetrics enforced and practised in that institution.

---

DR. RICHARD CURRAN, of Rochester, was elected Mayor of the Flower City, on Tuesday, March 7, 1892, by a majority which may reach 3,500 over the present incumbent, Mayor Carroll. Dr. Curran served during the entire civil war, having entered the service as an enlisted man, and when mustered out he was surgeon of the Ninth N. Y. Cavalry, a regiment raised principally in Chautauqua county. The election of Dr. Curran is another instance of the physician in politics, but no city can make a mistake when it elevates a physician of such sterling qualities as Dr. Curran to its chief magistracy.

Dr. John C. Fisher, late of the U. S. Marine Hospital Service, has located in Warsaw, N. Y., where he has established a sanitarium which is of the very first order. It is a most complete establishment of its kind, where salt baths can be had without the dangers of the surf. Dr. Fisher's sanitarium is located on a hillside, overlooking the beautiful Warsaw valley, and is one of the most picturesque spots in Western New York. Physicians who have patients requiring the rest and advantages of a sanitarium will find this one conducted on the strictest scientific and ethical principles. No physician need hesitate to send a patient there on account of fear that he will be supplanted or undermined.

---

Dr. James F. W. Ross, of Toronto, Ont., the leading abdominal surgeon in Canada, has recently paid a visit to Buffalo. Dr. Ross speaks in highest terms of many of the improved features of our growing city since his last visit, and promises that the time between this and his next will not be as long as before—in other words, that he will come and see us again soon. We shall always be pleased to welcome him.

---

Dr. John A. Ouchterlony, of Louisville, Ky., has been decorated by the King of Sweden with the Royal Order of Knight of the Polar Star. This knighthood is conferred only on those who have become famous as statesmen, scientists, great writers, etc., and was founded by King Frederick the I. of Sweden in 1738. Dr. Ouchterlony is of an illustrious Swedish family, distinguished for scientific research, and is recognized as one of the foremost American physicians. He is one of the honorary chairmen of the section on Practice of Medicine of the Pan-American Medical Congress, Professor of Practice of Medicine in the University of Louisville, and is regarded by the profession in that city as their most skilled practitioner and consultant on internal medicine. He is an accomplished linguist, a profound pathologist, and an expert diagnostician. Though this is a free American republic, we recognize the fitness of the royal honors that have been bestowed upon Sir John Ouchterlony, A. M., M. D.

---

Mrs. Gladstone's first article in the series of Hints from a Mother's Life, which she has written for *The Ladies' Home Journal*, will be printed in the April issue of that periodical.

